# *GATA3* amplification is associated with high grade disease in non-invasive urothelial bladder cancer but unrelated to patient prognosis

**DOI:** 10.1186/s12894-025-01704-y

**Published:** 2025-02-20

**Authors:** Henning Plage, Adrian Frericks, Sebastian Hofbauer, Kira Furlano, Sarah Weinberger, Florian Roßner, Simon Schallenberg, Sefer Elezkurtaj, Maximilian Lennartz, Andreas Marx, Henrik Samtleben, Margit Fisch, Michael Rink, Marcin Slojewski, Krystian Kaczmarek, Thorsten Ecke, Stefan Koch, Ronald Simon, Guido Sauter, Henrik Zecha, Joachim Weischenfeldt, Tobias Klatte, Sarah Minner, David Horst, Thorsten Schlomm, Martina Kluth

**Affiliations:** 1 Department of Urology, Charité– Universitätsmedizin Berlin, Corporate Member of Freie Universität Berlin, Humboldt-Universität zu Berlin and Berlin Institute of Health, Charitéplatz 1, 10117 Berlin, Germany; 2 Institute of Pathology, University Medical Center Hamburg-Eppendorf, Hamburg, Germany; 3 Institute of Pathology, Charité– Universitätsmedizin Berlin, Corporate Member of Freie Universität Berlin, Humboldt-Universität zu Berlin and Berlin Institute of Health, Berlin, Germany; 4 Department of Pathology, Academic Hospital Fuerth, Fuerth, Germany; 5 Department of Urology, University Medical Center Hamburg-Eppendorf, Hamburg, Germany; 6 Department of Urology, Marien Hospital Hamburg, Hamburg, Germany; 7 Department of Urology and Urological Oncology, Pomeranian Medical University, Szczecin, Poland; 8 Department of Urology, Helios Hospital Bad Saarow, Bad Saarow, Germany; 9 Department of Pathology, Helios Hospital Bad Saarow, Bad Saarow, Germany; 10 Department of Urology, Albertinen Hospital, Hamburg, Germany; 11 Biotech Research & Innovation Center (BRIC), University of Copenhagen, Copenhagen, Denmark

**Keywords:** GATA3, Urothelial bladder cancer, FISH, Prognosis

## Abstract

**Purpose:**

We aimed to assess the impact of GATA3 binding protein (GATA3) gene copy number alterations on tumor aggressiveness, patient prognosis, and GATA3 protein expression in a large urothelial bladder cancer cohort.

**Methods:**

A tissue microarray containing over 2,700 urothelial bladder cancers (pTa-pT4) was analyzed retrospectively using dual-labeling fluorescence in-situ hybridization (FISH) with probes for *GATA3* (10p14) and centromere 10. *GATA3* copy number gains were categorized as *GATA3* elevation (ratio *GATA3*/centromere ≥ 2/≤4), low-level amplification (ratio > 4/≤12), and high-level amplification (ratio > 12) and deletions were divided between homozygous and heterozygous.

**Results:**

*GATA3* copy number gain was detected in 9.9% of 2,213 interpretable tumors, including 2.0% with *GATA3* elevation, 3.2% with low-level amplification, and 4.7% with high-level amplification. The frequency of high-level amplification increased from pTa G2 low (0%) to pTa G3 tumors (12% [CI 0.07;0.21]; *p* < 0.0001 pTa G2 low vs. pTaG2 high) but decreased in advanced-stage carcinomas pT2-4 with 5.4% [CI 0.07;0.21] (*p* < 0.0001, pTa vs. pT2-4). In muscle-invasive carcinomas, *GATA3* amplification was not linked to tumor aggressiveness or patient survival. Overall, no homozygous *GATA3* deletion was detected and heterozygous *GATA3* deletion was only observed in 1.1%; of 1,432 pT2-4 tumors without any association to cancer progression. While *GATA3* copy number was significantly correlated with GATA3 expression (*p* < 0.0001), the relationship was not strong. Only 2.3% of *GATA3*-negative cancers had a deletion, and 42.1% of strong GATA3-expressing cancers exhibited high-level amplification.

**Conclusion:**

High-level *GATA3* amplification is common in urothelial bladder cancer and correlates with grade progression in pTa tumors, while *GATA3* deletion is rare. Neither amplification nor deletion appears to be the primary driver of GATA3 expression dysregulation.

**Clinical trial number:**

Not applicable.

**Supplementary Information:**

The online version contains supplementary material available at 10.1186/s12894-025-01704-y.

## Introduction

GATA binding protein 3 (GATA3), a zinc finger transcription factor, plays a pivotal role in regulating cell differentiation and tissue-specific gene expression. Initially recognized for its role in T-cell development and differentiation in breast tissue, GATA3 has emerged as a key marker in differentiation in urothelium [1]. Consequently, GATA3 immunohistochemistry (IHC) is frequently utilized in diagnostic surgical pathology to differentiate primary or metastatic urothelial bladder cancer (UBC) from morphologic duplicates. GATA3 expression is considered a hallmark of the luminal subgroup of UBC which is characterized by a favorable patient prognosis [2, 3]. While non-invasive UBC, predominantly classified as luminal tumors, exhibit a universally high expression profile of GATA3, loss of GATA3 expression is observed in advanced-stage disease [3]. One potential mechanism for GATA3 - located at chromosome 10p14 - dysregulation involves alterations in gene copy number. Several earlier studies had suggested that both *GATA3* gene amplification and deletion may occur at significant frequency in UBC [4–11]. Copy number gains of the 10p14 region have been described to occur in 2–50% of UBC in studies analyzing up to 476 cancers [4–9]. Two studies identified 10p14 amplification in 2% of 90 cases and 10% of 476 pT1 to pT4 carcinomas, respectively [8, 9]. Four studies showed 10p14 deletions in 0.2–38% of up to 476 pT2-pT4 carcinomas by classical or array-based comparative genomic hybridization (CGH, aCGH), and loss of heterozygosity (LOH) analysis [4, 10, 11]. Notably, several of these studies found a significant association between 10p14 copy number gains and unfavorable tumor features [4–6, 8].

In the context of optimizing treatment decisions and enabling more aggressive therapeutic strategies for high-risk patients, a better prediction of individual cancer aggressiveness and patient prognosis is urgently needed. Achieving this requires a deeper understanding of the molecular features driving UBC progression. To learn more about the prevalence and clinical relevance of *GATA3* (10p14) chromosomal alterations in UBC as well as its role in aberrant GATA3 expression, the *GATA3* copy number status was analyzed in more than 2,700 UBC in a tissue microarray (TMA) format by fluorescence in-situ hybridization (FISH). The results were compared with clinicopathological parameters of disease progression, patient outcome, and GATA3 expression data obtained by IHC.

## Materials and methods

### Tissue microarrays (TMA)

The TMAs used in this study were first employed in a study on the prognostic role of GATA3 expression in UBC [3]. The TMAs contained one sample each from 2,710 UBC archived at the Institute of Pathology, University Hospital Hamburg, Germany, Institute of Pathology, Charité Berlin, Germany, Department of Pathology, Academic Hospital Fuerth, Germany, or Department of Pathology, Helios Hospital Bad Saarow, Germany, and/or treated at Department of Urology, University Hospital Hamburg, Germany, Department of Urology, Charité Berlin, Germany, Department of Urology, Helios Hospital Bad Saarow, Germany, Department of Urology, Albertinen Hospital, Hamburg, Germany, and Department of Urology and Urological Oncology, Pomeranian Medical University, Szczecin, Poland. Patients at each center were treated according to the guidelines at the time. In brief, patients with pTa disease underwent a transurethral bladder tumor resection with or without postoperative or adjuvant instillation therapy. 1,826 patients with pT2-pT4 disease were treated by radical cystectomy or transurethral bladder tumor resection. Available histopathological data including tumor stage (pT), grade, status of venous (V) and lymphatic (L) invasion, and lymph node status (pN) are shown in Table 1. Clinical follow up data were available from 709 patients with pT2-4 carcinomas treated by cystectomy (overall survival time: median: 13 months, range: 1-176 months, recurrence-free survival: median: 11 months, range 1–75 months, cancer-specific survival: median: 14 months, range 1–77 months). Follow-up was conducted by the treating physicians of each center through calls to patients and outpatient doctors, clinical registers, and clinical visits. IHC data on GATA3 expression were from a previous study [3]. All tissues were fixed in 4% buffered formalin and then embedded in paraffin. The TMA manufacturing process has previously been described in detail [12, 13]. In brief, one tissue spot (diameter: 0.6 mm) per patient was used. The use of archived remnants of diagnostic tissues for TMA manufacturing, their analysis for research purposes, and patient data were according to local laws (HmbKHG, § 12) and analysis had been approved by the local ethics committee (Ethics commission Hamburg, WF-049/09). A Clinical trial number is not applicable. All work has been carried out in compliance with the Helsinki Declaration.

**Table 1:** Patient cohort

	study cohort on TMA (*n*=2710)
**follow up**	
months	709
mean	24.5
median	13
**tumor stage**	
pTa	887 (39.2%)
pT2	462 (20.4%)
pT3	615 (27.2%)
pT4	298 (13.2%)
**tumor grade**	
G2	820 (30.6%)
G3	1858 (69.4%)
**lymphnode metastasis**	
pN0	734 (62.0%)
pN+	449 (38.0%)
**resection margin**	
R0	595 (80.6%)
R1	143 (19.4%)
**lymphatic invasion**	
L0	275 (49.5%)
L1	281 (50.5%)
**venous invasion**	
V0	450 (74.4%)
V1	155 (25.6%)

Percent in the column “study cohort on TMA” refers to the fraction of samples across each category. Numbers do not always add up to 2,710 in the different categories because of cases with missing data

### Fluorescence in-situ hybridization (FISH)

**Fig. 1:**
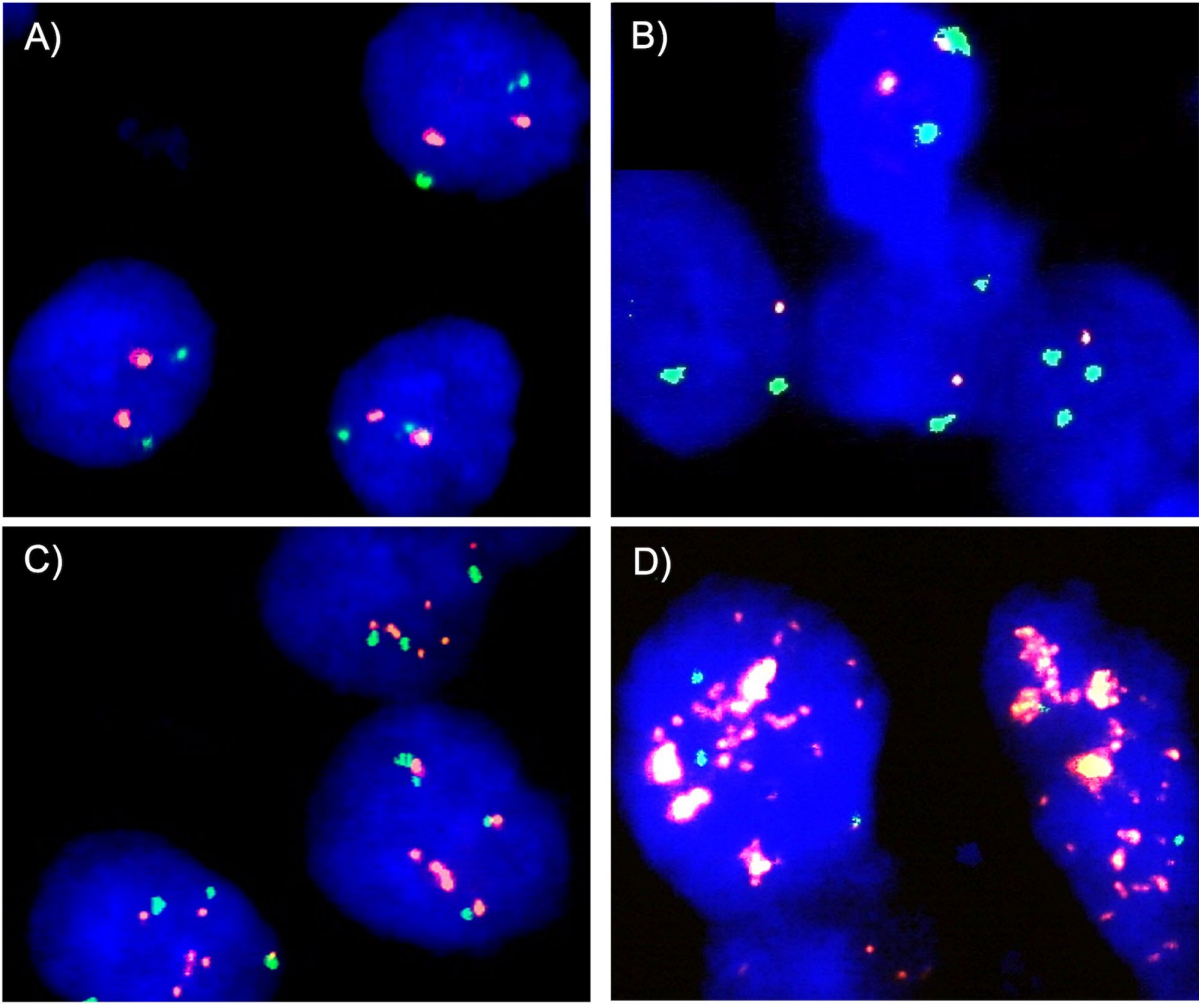
Examples of *GATA3*copy numbers by fluorescence in-situhybridization. **A**) non-altered *GATA3* copy number status with two orange *GATA3* signals and two green centromere 10 signals, **B**) *GATA3* heterozygous deletion with one orange *GATA3* signal and two green centromere 10 signals, **C**) elevated *GATA3* copy number status with a *GATA3*/centromere 10 ratio ≥ 2 and < 4, and **D**) high-level *GATA3* amplification with a *GATA3*/centromere 10 ratio ≥ 12

Five micrometer TMA sections were deparaffinized with xylol, rehydrated through a graded alcohol series and exposed to heat-induced denaturation for 10 minutes in a water bath at 99°C in P1 pretreatment solution (Agilent Technologies, Santa Clara, CA, USA; #K5799). For proteolytic treatment, slides were added to VP2000 protease buffer (Abbott, North Chicago, IL, USA; #2J.0730) for 200 minutes at 37°C in a water bath. A commercial FISH probe kit containing both, gene specific *GATA3* / 10p14 and corresponding centromere 10 probes, were utilized for copy number detection of *GATA3* (*GATA3* gene locus and centromere 10; self-designed, Empire Genomics, Buffalo, NY, USA). Hybridization was performed overnight at 37°C in a humidified chamber. Posthybridization washes were done according to the manufacturer’s direction (Agilent Technologies, Santa Clara, CA, USA; #K5799). Nuclei were counterstained with 125 ng/ml 4’,6-diamino-2-phenylindole in antifade solution (Biozol; Eching, Germany; #VEC-H-1200). Copy numbers of *GATA3* and centromere 10 were estimated for each tissue spot as follows. Presence of fewer *GATA3* signals than centromere 10 signals in at least 60% of all tumor nuclei in a tumor spot was considered a heterozygous deletion. Absence of *GATA3* signals in the presence of centromere 10 signals in all tumor nuclei and presence of *GATA3* and centromere 10 signals in normal cell nuclei was considered a homozygous deletion. Presence of more *GATA3* signals as centromere 10 signals was considered a *GATA3* gain if the ratio from *GATA3* to centromere 10 was ≥ 2. For statistical analyses tumors were divided into three groups. *GATA3* elevated with *GATA3*/centromere 10 ratio ≥ 2 and < 4, *GATA3* low-level amplification with *GATA3*/centromere 10 ratio ≥ 4 and < 12, and *GATA3* high-level amplification with *GATA3*/centromere 10 ratio ≥ 12. All other tumors were considered as non-altered. Figure 1 gives examples of different *GATA3* copy number status. Tissue spots without any detectable *GATA3* signals in all tumor and normal cell nuclei were excluded from the analysis because of a lack on an internal control for successful hybridization.

### Statistics

Statistical calculations were performed with JMP17^®^ software (SAS^®^, Cary, NC, USA, version 17). Contingency tables and the chi²-test were performed to search for associations between *GATA3* copy number status and GATA3 IHC as well as tumor phenotype. Survival curves were calculated according to Kaplan-Meier. The Log-Rank test was applied to detect significant differences between groups. A p-value of ≤ 0.05 was considered as statistically significant.

## Results

### Technical issues

The *GATA3* copy number status was detectable in 2,213 (81.7%) of 2,710 UBC in our FISH analysis. Reasons for non-analyzable tissues (497 spots; 18.3%) were insufficient FISH, lack of tissue spots, and absence of unequivocal cancer cells in the TMA spot.

### *GATA3* copy number status in urothelial bladder cancers

**Fig. 2:**
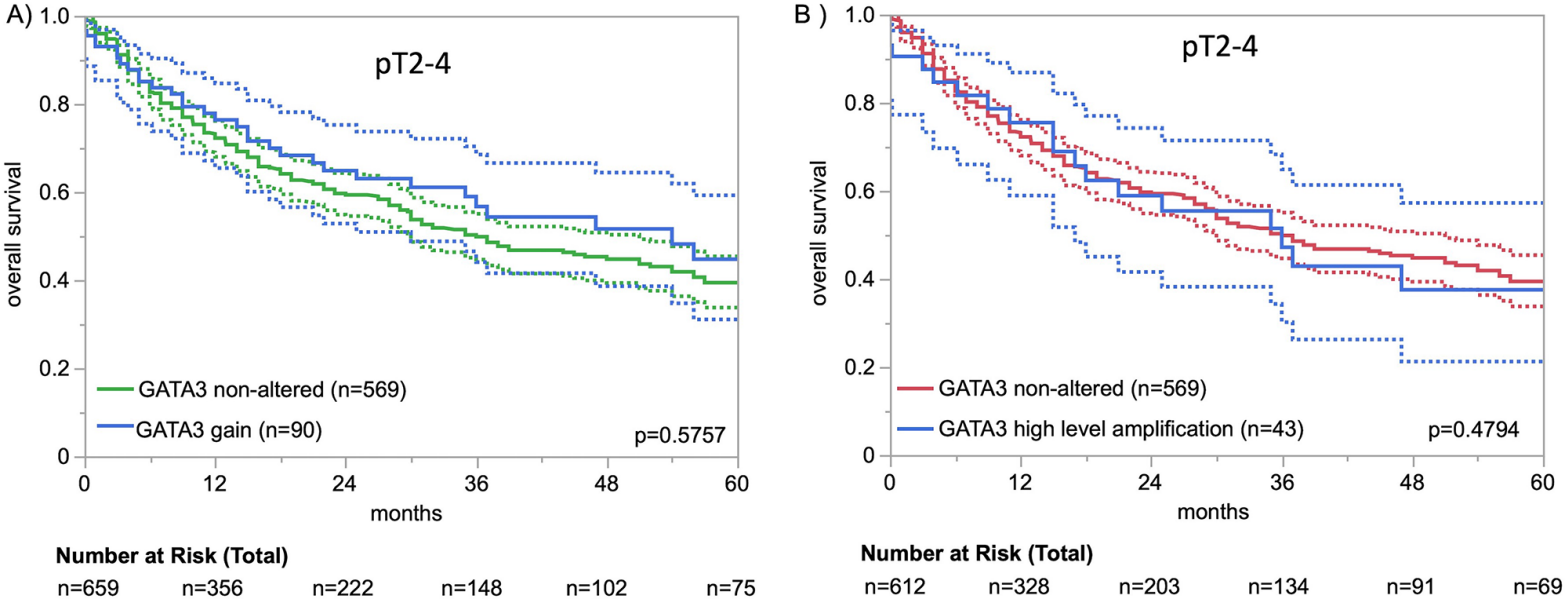
*GATA3* copy number status and patient survival in muscle invasive pT2-4 UBC. **A**) non-altered vs. gain (ratio *GATA3*/centromere 10 ≥ 2, and **B**) non-altered vs. high-level amplification (ratio *GATA3*/centromere 10 ≥ 12). The dashed line represents the lower and upper 95% confidence interval

An increased number of *GATA3* gene copies were detectable in 218 (9.9%) of the 2,213 analyzable carcinomas, including 44 (2.0%) with *GATA3* elevation, 71 (3.2%) with low-level amplification, and 103 (4.7%) with high-level amplification. The increase of *GATA3* copy numbers was associated with higher grades in non-invasive UBC. For example, the fraction of *GATA3* high-level amplificated carcinomas increased from pTa G2 low (0%), to pTa G2 high (2.4%), and pTa G3 (12.1%; *p* < 0.0001). The fraction of tumors with *GATA3* high-level amplification was higher in pT2-4 carcinomas (5.4%) than in (all) pTa tumors (pTa 2.4%; *p* < 0.0001 for pTa vs. pT2-4; Table 2) but it was lower than in pTa G3 tumors (pTa G3 12.1%; *p* < 0.0001 for pTa G3 vs. pT2-4). In pT2-4 carcinomas, *GATA3* copy numbers were unrelated to parameters of tumor aggressiveness and patient prognosis (Table 2; Fig. 2, Supplementary Fig. 1). A *GATA3* deletion was not seen in any of 539 pTa tumors but it was detectable in 16 (1.1%) of the 1,432 analyzable pT2-4 carcinomas and it was always heterozygous. *GATA3* deletion was unrelated to clinicopathological parameters in pT2-4 carcinomas (Table 2).

**Table 2:** *GATA3* copy number status and cancer phenotype

		GATA3 copy number status					
	n	deletion	non-altered	elevated	low level amplification	high level amplification	
All cancers	2213	0.7	89.7	2.0	3.0	4.6	
pTa G2 low	329	0.0	100.0	0.0	0.0	0.0	*<0.0001
pTa G2 high	127	0.0	96.9	0.0	0.8	2.4	**<0.0001
pTa G3	83	0.0	81.9	3.6	2.4	12.1	
pT2	418	1.0	85.2	1.9	6.2	5.7	*0.2652
pT3	559	1.4	87.1	3.0	3.4	5.0	**0.1462
pT4	266	1.1	89.9	2.3	2.3	4.5	***0.8057
G2	19	0.0	89.5	0.0	0.0	10.5	*0.5920
G3	350	0.3	88.3	2.6	3.1	5.7	
pN0	34	0.0	82.4	2.9	5.9	8.8	*0.8600
pN+	2	0.0	100.0	0.0	0.0	0.0	
R0	540	1.1	87.8	3.2	4.1	3.9	*0.8298
R1	125	2.4	88.0	2.4	3.2	4.0	
L0	248	1.6	86.7	3.6	4.0	4.0	*0.9745
L1	254	1.6	88.6	3.2	3.2	3.5	
V0	405	1.5	87.9	2.7	3.7	4.2	*0.8215
V1	138	2.9	86.2	3.6	2.9	4.4	

Abbreviations: pT: pathological tumor stage, G: Grade, pN: pathological lymph node status, R: resection margin status, L: lymphatic invasion, V: venous invasion; *only in pT2-4 urothelial carcinoma, **non-altered vs. gain (elevated, low and high level amplification), ***non-altered vs. deletion

### *GATA3* copy number status vs. GATA3 immunostaining

**Fig. 3:**
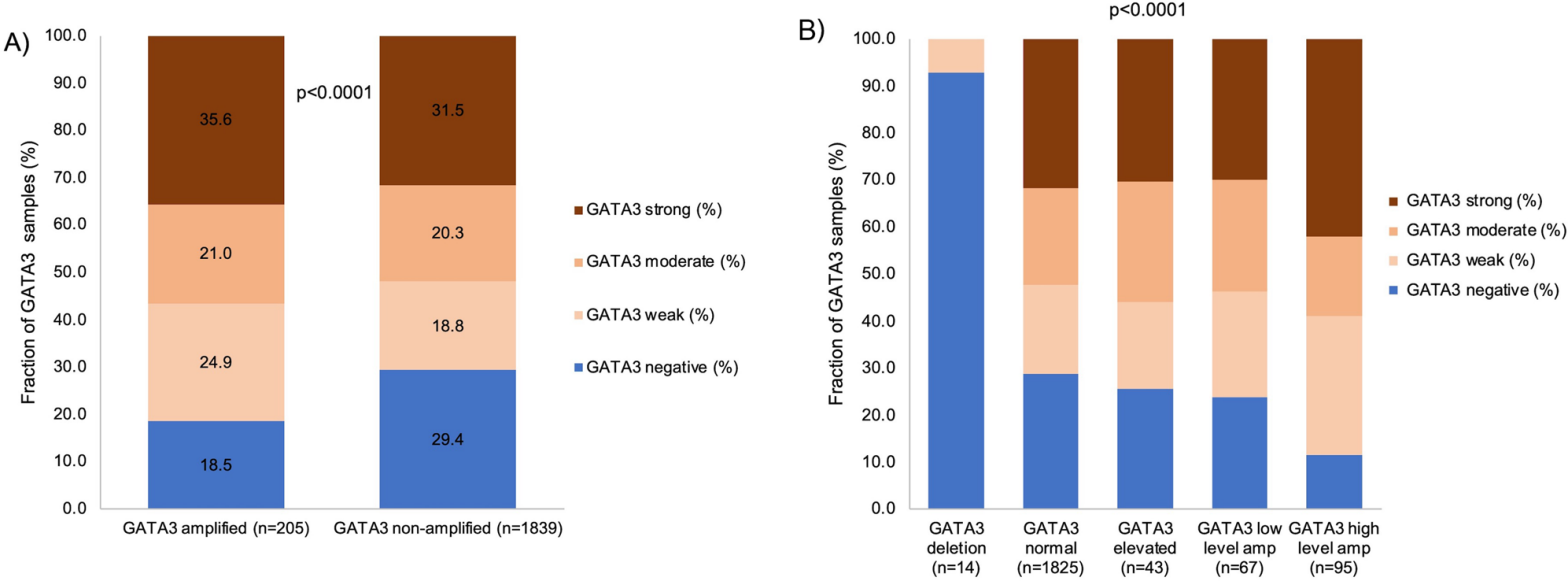
Association *GATA3*copy number status vs. GATA3 immunostaining. **A**) GATA3 amplified (ratio GATA3 signal/centromere 10 signal ≥ 2) vs. GATA3 non-amplified (ratio GATA3 signal/centromere 10 signal < 2), **B**) detailed GATA3 copy number status (GATA3 deletion with GATA3/centromere 10 ratio < 0.5, GATA3 normal with GATA3/centromere 10 ratio ≥ 0.5 and < 2, GATA3 elevated with GATA3/centromere 10 ratio ≥ 2 and < 4, GATA3 low-level amplification with GATA3/centromere 10 ratio ≥ 4 and < 12, and GATA3 high-level amplification with GATA3/centromere 10 ratio ≥ 12

A comparison of *GATA3* FISH and IHC data revealed a statistically significant but not a very close association between *GATA3* copy number status and *GATA3* IHC results in 2,044 UBC (*p* < 0.0001; Fig. 3). Tumors with increased *GATA3* copy numbers were more often positive by GATA3 IHC (81.5%) than carcinomas with non-altered *GATA3* copy number status (71.1%; *p* < 0.0001; Fig. 3A) and cancers with *GATA3* high-level amplifications showed more often strong GATA3 immunostaining (42.1%) than carcinomas without high-level amplifications (29.9–31.8%; *p* = 0.0002, Fig. 3B) but 11.6% of cancers with high-level GATA3 amplification were GATA3 IHC negative. Although most carcinomas with *GATA3* deletions were negative by GATA3 IHC (92.9%), a *GATA3* deletion was only found in 2.3% of 578 GATA3 IHC negative cancers.

## Discussion

In this study, *GATA3* copy number gains (*GATA3*/centromere 10 ratio > 2) were found in 9.9% of all analyzable UBCs. This frequency is in the lower range of previous studies which reported 2–50% *GATA3* copy number gains in cohorts of 30 to 476 UBC by using CGH and aCGH as a method [4–9]. However, our rate of 4.7% high-level and 3.2% low-level *GATA3* amplifications is in line with two earlier studies showing *GATA3* amplifications in 2% of 90 and 10% of 476 analyzed UBC [8, 9]. Differences in the rate of copy number alteration between studies is mainly due to the use of different methods (isolated DNA vs. in-situ) and cut-off values [14]. FISH is considered the gold standard for copy number changes because gene copy numbers can exactly be determined on a cell-by-cell basis. This makes the method independent of possible “contaminations” by DNA from non-neoplastic cells as well as copy number variations based on polysomy or aneusomy and DNA replication during cell cycle progression which is often a problem in methods using isolated DNA for gene copy number analysis such as CGH, aCGH or LOH analysis. FISH has so far not been utilized for *GATA3* amplification analysis in urothelial carcinomas.

The marked increase of *GATA3* high-level amplifications from pTa G2 low-grade (0%) to pTa G2 high-grade (2.4%), and pTa G3 (12.1%) is consistent with a continuous increase of genomic instability during cellular dedifferentiation which parallels grade progression in non-invasive UBC [15]. The lower rate of *GATA3* high-level amplifications (5.4%) in pT2-4 than in pTa G3 UBCs can be explained by the particular evolution of pTa UBCs in vivo. Non-invasive urothelial tumors can recur over many years and even decades [16]. Previous studies assume that multiple recurrent non-invasive urothelial cancers are often caused by cell dissemination from the original tumor [17, 18]. Particular, high-risk tumors like pTa G3 can thus continuously accumulate genomic alterations over a very long period with increasing dedifferentiation. Thus GATA3, which is known to have a pivotal role in urothelial differentiation, might be more affected in contrast to muscle-invasive tumors. Tumor cells with the ability to grow invasively have less time for further dedifferentiation because these tumors are either surgically removed in a timely manner or the patient succumbs to invasive UBCs.

That neither *GATA3* copy number gains nor high-level amplifications which are both features of cellular dedifferentiation were associated with unfavorable histologic parameters or poor prognosis is muscle-invasive urothelial carcinoma is in contrast to two earlier studies showing an association between *GATA3* gain and presence of metastasis and poorer patient survival [4, 8]. However, it appears to be a hallmark of urothelial carcinoma of the urinary bladder that typical progression markers such as p53 alterations [19], HER2 overexpression [20], and MYC alterations [21] are not prognostic in these tumors. In agreement with these disappointing findings, it has been recommended in all WHO classifications of genitourinary carcinomas since 2004 to avoid histologic grading in pT2-4 tumors because of its lack of clinical relevance [22]. In earlier studies on the same set of tumors, we had also failed to find a prognostic role of 17p (*TP53*) deletions (MS in revision) and *CCND1* amplifications (MS in revision).

Our data do not support a major regulatory role of *GATA3* copy number gains for GATA3 expression in urothelial carcinoma although there was a significant statistical association between *GATA3* gene copy number and expression. That only 40% of *GATA3* high-level amplificated carcinomas showed strong GATA3 immunostaining while 12% were entirely GATA3 negative demonstrates that *GATA3* amplification is neither required nor sufficient to induce strong GATA3 expression in these tumors. The complete absence of GATA3 immunostaining in 11 of 95 carcinomas with high-level *GATA3* amplification is consistent with data showing that only expressed proteins can be overexpressed in case of gene amplification [23]. That only 6% of cancers with strong GATA3 staining harbored a high-level amplification further demonstrates that gene copy number gains are not the major cause for increased GATA3 expression. Other known mechanism for up-regulation of GATA3 expression include transcriptional activation by cancer-associated pathways as the NOTCH1 and NFkB pathway [24] as well as *GATA3* promoter hypomethylations [25].

GATA3 is expressed in normal urothelium but its expression is reduced or completely lost in 2% of pTa and 40% of pT2-4 UBC [3]. Our data do not support a major role of *GATA3* deletion for GATA3 expression loss. Our *GATA3* deletion rate of 1.1% was in the lower range of previous studies reporting 0.2–38% *GATA3* deletions obtained by methods which are more dependent on tumor cell purity and thresholds for defining deletions such as CGH, aCGH, and LOH analysis [9–11]. That all 16 *GATA3* deletions of this study were found in pT2-4 UBC is - in principle - in line with earlier data demonstrating that GATA3 expression loss preferably occurs in advanced invasive tumors [3]. However, that 97.7% of all GATA3 negative UBCs were non-deleted demonstrates that *GATA3* deletion is not a major mechanism for GATA3 down-regulation in UBCs. Other known mechanisms for down-regulation or loss of GATA3 expression include several frameshift, truncation, or splice site gene mutations [26, 27] and epigenetic hypermethylation of the *GATA3* promotor region [28].

The study has several limitations. Despite employing a substantial tumor cohort for the tissue microarray (TMA), the sample size remains insufficient to detect significant events. Notably, with a high-level GATA3 amplification rate of only 5%, there are merely 103 tumors available to assess differences in tumor phenotype and patient prognosis. Additionally, a larger cohort than the current 709 patients with follow-up data would be preferable. The retrospective nature of the study, encompassing patients from 2003 to 2021, introduces variability in the availability and consistency of clinical data. This is particularly evident in the incomplete data regarding systemic therapies (adjuvant or neoadjuvant chemotherapy in pT2-4 tumors) and intravesical therapies for non-invasive disease.

In summary, *GATA3* amplification is a common event which parallels tumor cell dedifferentiation in urothelial neoplasms, especially in non-invasive pTa tumors. *GATA3* deletions are very rare in urothelial cancer and unrelated to the tumor phenotype. *GATA3* copy number changes are not critical drivers for deregulated GATA3 expression in urothelial carcinomas.

## Electronic supplementary material

Below is the link to the electronic supplementary material.

Supplementary Material 1

## Data Availability

All data generated or analyzed during this study are included in this published article.

## Abbreviations

aCGH: array-based Comparative Genomic Hybridization
CGH: Comparative Genomic Hybridization
CI: Confidence Intervall
FISH: Fluorescence In-Situ Hybridization
GATA3: GATA binding protein 3
IHC: Immunohistochemistry
LOH: Loss Of Heterozygosity
TMA: Tissue Microarray
UBC: Urothelial Bladder Cancer
