# Supplementary figures and images for: GATA3 amplification is associated with high grade disease in non-invasive urothelial bladder cancer but unrelated to patient prognosis
Source: BMC Urol. 2025 Feb 20;25:37. doi: 10.1186/s12894-025-01704-y (PMC11841250; doi:10.1186/s12894-025-01704-y)

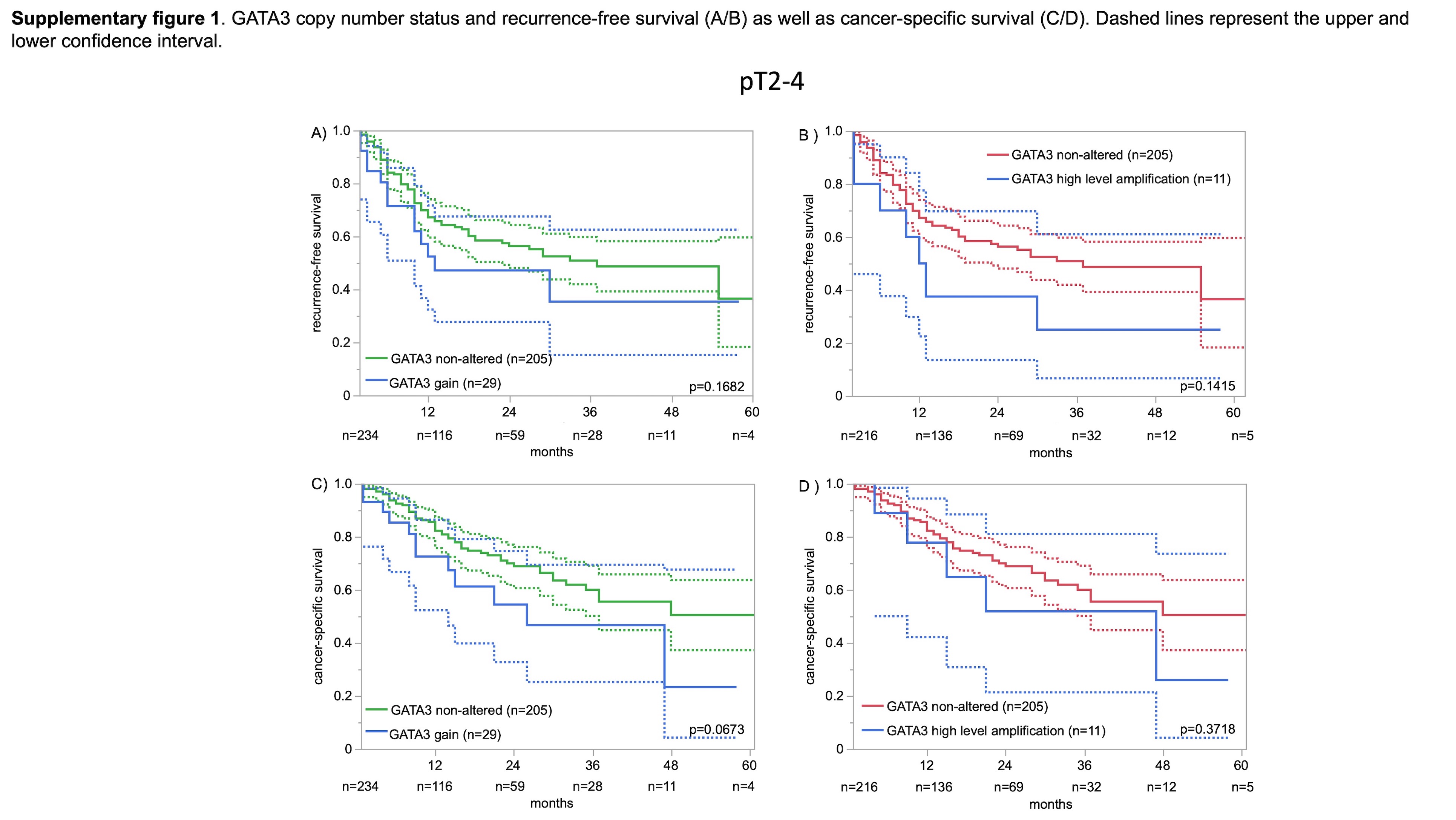

Supplement: Supplementary file 1 — Supplementary Material 1 [file 12894_2025_1704_MOESM1_ESM.jpg]
